# Four-rod Instrumentation for Treatment of Charcot Spinal Arthropathy Causing Autonomic Dysreflexia: Case Report and Literature Review

**DOI:** 10.7759/cureus.850

**Published:** 2016-10-27

**Authors:** Stephanie Zyck, Gentian Toshkezi, John Pizzuti, Satya Marawar

**Affiliations:** 1 Neurosurgery, SUNY Upstate Medical University; 2 Internal Medicine, St. Joseph's Medical Center; 3 Orthopedic Spine Department, Syracuse Veterans Affairs Hospital

**Keywords:** spine, charcot arthropathy, autonomic dysreflexia

## Abstract

Late complications of spinal cord injury can include Charcot arthropathy, in which spinal instability occurs as a result of repetitive trauma in the insensate spine. In rare cases, this can present as autonomic dysreflexia. We present the case of a 60-year-old man with longstanding C6 quadriplegia who presented with six months of hypertension, diaphoresis and dizziness. After an extensive workup, the patient's symptoms were attributed to autonomic dysreflexia in the setting of spinal instability from Charcot spinal arthropathy. Computed tomography (CT) and magnetic resonance imaging (MRI) revealed instability with degenerative changes at L1-L2. We present our case with a literature review to discuss management of this uncommon situation.

The patient underwent posterior fusion and instrumentation from T8-L5 with four rods, alternating screws and crosslinks with a good reduction and solid stabilization of the spine. Postoperatively, the patient experienced immediate relief of all symptoms. Our case demonstrates effective surgical treatment for Charcot spinal arthropathy causing autonomic dysreflexia. Stabilization with instrumentation and fusion of underlying Charcot spinal arthropathy removed the trigger of the autonomic dysreflexia and alleviated our patient's symptoms.

## Introduction

Neuropathic spinal arthropathy, or Charcot spine, occurs when the destruction of afferent sensory fibers allows recurrent minor trauma to go unnoticed, leading to the mechanical degeneration of facet joints [1-5]. The loss of these protective sensory fibers is most commonly caused by spinal cord injury, but also can occur as a result of diabetic neuropathy, tertiary syphilis, syringomyelia, anesthetic leprosy, and congenital absence of pain syndrome [1-2, 4, 6-7]. Insensitivity to pain in quadriplegic or paraplegic patients can allow the Charcot joint to progress to a point of hypermobility and spinal instability before being recognized. Charcot arthropathy most often presents with progressive spinal deformity, back pain, neurologic changes, and audible cracking heard during change in position [1, 4].

Autonomic dysreflexia, one of the more rare complications of Charcot spine, is a potentially life-threatening acute reaction of the autonomic nervous system to overstimulation that is characterized by hypertension, concurrent bradycardia, diaphoresis, flushing, and anxiety [8]. Patients with an injury above T6 (location of splanchnic sympathetic outflow) are particularly vulnerable to autonomic dysreflexia due to the loss of supraspinal control of spinal sympathetic output below the level of the lesion [1, 4-5, 8]. After the spinal shock subsides, hyperreflexic sympathetic activity can occur such that a nonspecific stimulus below the level of the lesion can provoke uncontrolled sympathetic output with resultant cardiovascular effects [8]. Previously, there have been some limited reports of patients with Charcot spine who have presented with autonomic dysreflexia [4-5].

Surgical stabilization of unstable segments is a mainstay of treatment, though, in select cases, conservative management with long-term recumbency and external support has also been utilized. Surgical treatment frequently involves extensive debridement, anterior and posterior fusion, and posterior instrumentation [1, 7]. In this case we utilized a four-rod construct as described by Kelly and Shen [9-10]. To our knowledge, we are the first to describe the application of this surgical technique in a patient with Charcot arthropathy presenting with autonomic dysreflexia.

Informed consent was obtained from the patient for this procedure.

## Case presentation

A 60-year-old male with a history of C6 American Spinal Injury Association (ASIA) A quadriplegia 41 years prior, presented with the new complaint of episodes of spiking hypertension, diaphoresis, and dizziness. This had occurred on a daily basis for six months and was brought on by any change in trunk position. His systolic blood pressure was noted to rise up to 200 mmHg during these episodes. While the patient had no remaining sensory or motor function below the C6 level and no remaining voluntary bowel or bladder function, the patient reported that due to the high frequency of these episodes he had completely lost any remaining quality of life.

**Figure 1:**
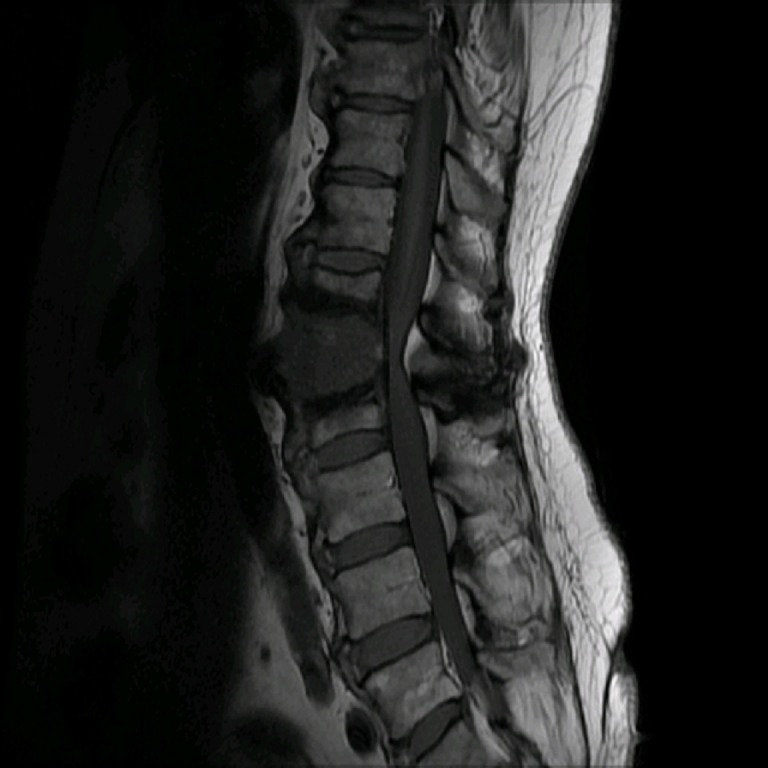
Sagittal T1 MRI This preoperative sagittal T1 weighted MRI without contrast reveals expansion of L1-2 disc space with edema and erosion of adjacent endplates.

**Figure 2:**
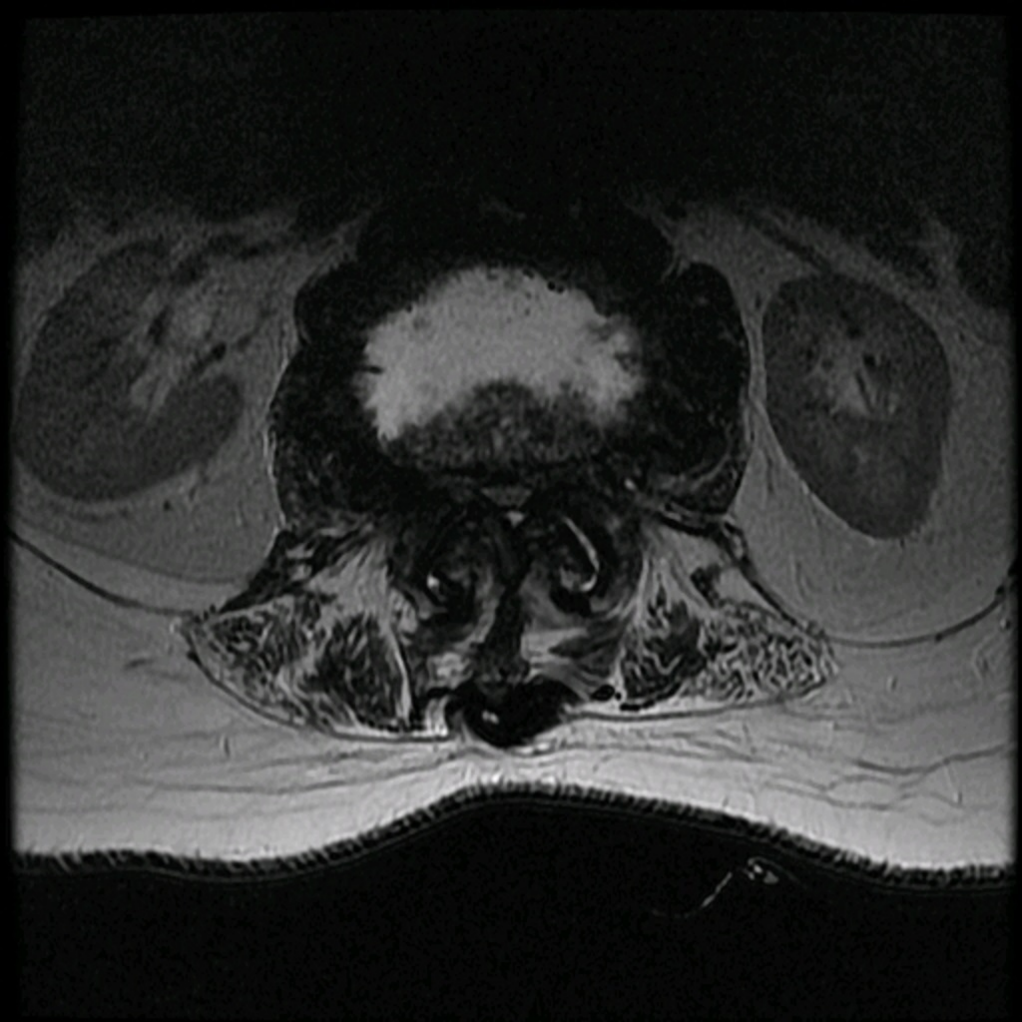
Axial T2 MRI This preoperative axial T2 MRI demonstrates severe spinal and neural foraminal stenosis at the L1-2 level.

CT of the lumbar spine revealed an enlarged L1-2 disc space with over 50% erosion of the adjacent endplates, along with vacuum disc. Severe spinal canal and neural foraminal narrowing was also seen with multiple large bridging osteophytes. The rest of the thoracolumbar spine had the appearance of diffuse idiopathic skeletal hyperostosis and was fused. An MRI of the lumbar spine revealed edema of the L1-2 endplates as well as some fluid in the disc space, as shown in Figures 1-2.

A needle aspiration of the L1-2 disc space was performed to rule out infection and these results were negative. As extensive workup ruled out other causes, it was determined that the patient’s clinical picture and imaging findings were most consistent with autonomic dysreflexia secondary to Charcot’s arthropathy of the lumbar spine.

The patient underwent posterior instrumentation from T8 to L5 with four-rod fixation and partial L1 and L2 corpectomy with extracavitary approach. The patient was positioned prone and the incision was made midline from T8 down to the S1 level. Extension to S1 was originally planned, but, while performing the dissection, it was noted that, in order to expose S1, the incision would need to be extended through unhealthy areas of the skin near the anal opening. Due to concern for difficult wound healing, the decision was made to limit instrumentation to the L5 level. A four-rod construct was utilized, which would involve using medial as well as lateral entry points for the pedicle screws with the goal of connecting two interlinked rods on each side. As per our surgical planning, we proceeded with pedicle screw instrumentation. Lateral and medial entry points for pedicle screws were alternated. The lateral entry point was at the junction of the lateral border of the superior facet along the line bisecting the transverse process. The medial entry point for the pedicle screw was along the medial border of the superior facet along the line bisecting the transverse process. Once pedicle screws were placed and confirmed with fluoroscopy, the right lateral and then medial titanium rods were contoured, placed, and fixed using set screws. 

An extracavitary approach was then performed and partial corpectomy at L1-2 was performed from the left side. Transverse processes of L1 and L2 were exposed and then removed. Further medial dissection was performed to expose the exiting nerve root. The lateral portion of the L1-2 facet was removed using a drill. Once the L1 root was isolated, it was tied off and transected. Following this, further removal of the L1-2 facet and lateral part of the lamina was done to expose the dura. Ligamentum flavum was removed. Once the dura was exposed, it was retracted medially. This allowed posterolateral extracavitary access to the L1-2 disc space and vertebral bodies.

**Figure 3:**
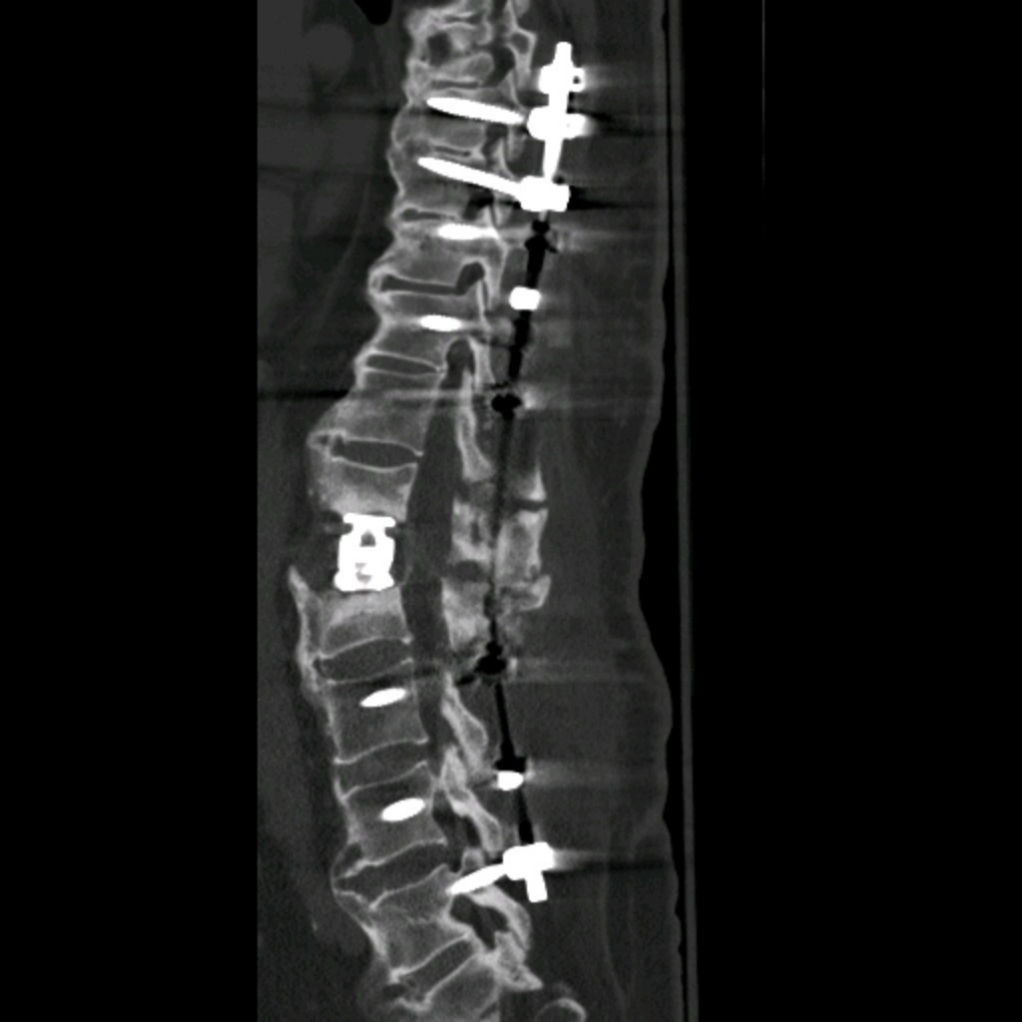
Sagittal postoperative CT This sagittal view of the postoperative CT of the thoracolumbar spine shows intact titanium rods and screws spanning from T8 to L5 with placement of an expandable titanium cage at L1-2.

**Figure 4:**
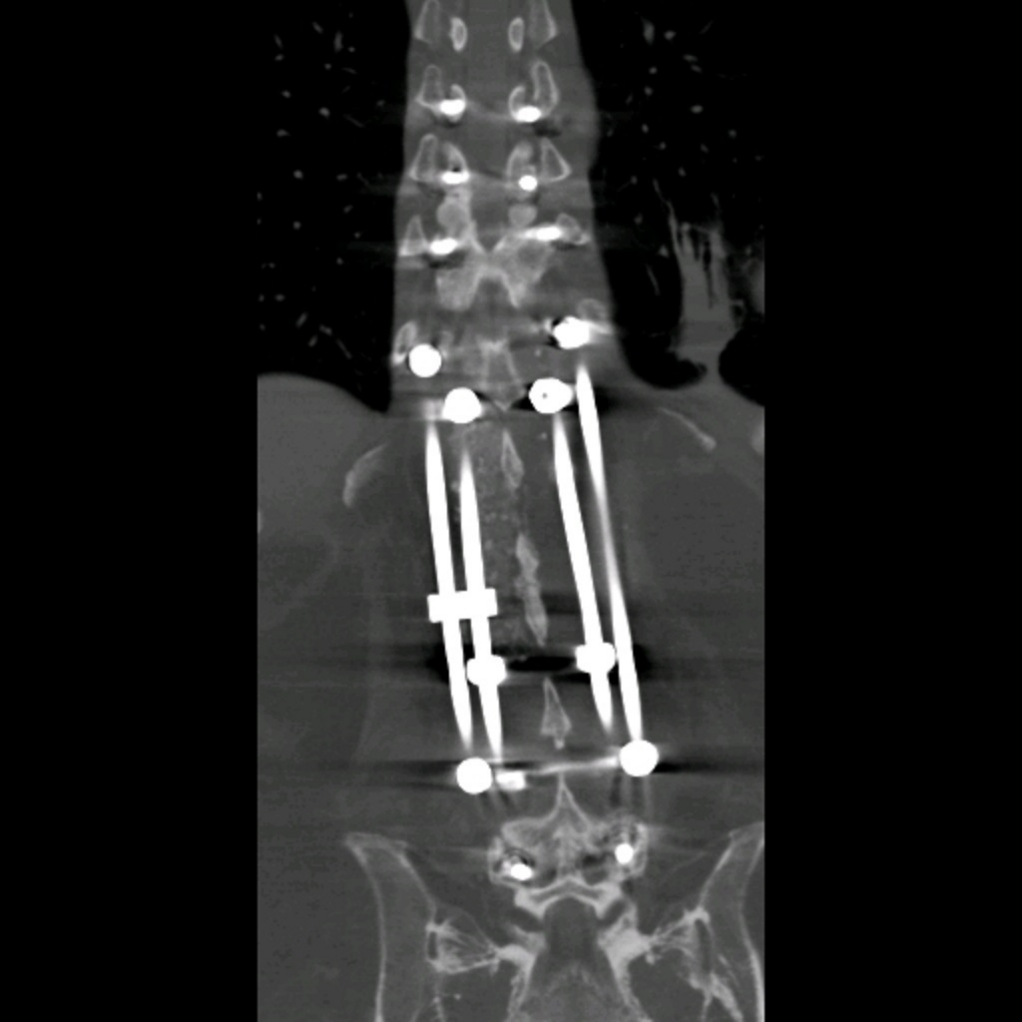
Coronal postoperative CT This coronal view of the postoperative CT of the thoracolumbar spine demonstrates the four-rod construct that was utilized. Medial and lateral entry points for pedicle screw placement were alternated from T8-12 and L3-5, as shown, prior to rod placement and utilization of rod-to-rod connectors.

We then entered the area of pathology at L1-2. There was a large void in the L1-2 disc space. The endplates were decorticated and samples were sent for pathology; these were negative for tumor or infection. An expandable titanium cage was then placed and filled with bone morphogenetic protein. Following this, the pedicle screws on the left side were connected with the medial and lateral rods which were adequately sized and contoured. These rods were again interlinked with a rod-to-rod connector. Posterolateral fusion was done across L1-2 on the right side with cancellous bone allograft and bone morphogenetic protein. The reduction and stabilization of the spine was confirmed through posterior x-rays and the postoperative CT, as shown in Figures 3-4.

Postoperatively, the patient had an uncomplicated course and he immediately had full recovery of his symptoms related to autonomic dysreflexia. At a six-month follow-up, the patient continued to have full relief of his symptoms of autonomic dysreflexia.

## Discussion

The development of Charcot joints stems from sensory deprivation allowing repetitive trauma to go unnoticed by the subject. Stretching of ligamentous and capsular structures then fails to stimulate the oppositional protective contraction and stabilization of the spine. Over time, this process results in the destruction of articular cartilage and joint capsules, sclerosis, and eventually osteophytes that bridge the vertebral bodies [3, 6-7]. Extensive laminectomies with removal of posterior ligamentous elements may cause increased instability of affected joints and potentiate the neuropathological processes in Charcot arthropathy [3-5].

Patients with spinal cord injury above the splanchnic outflow at T6 are at an increased risk of suffering autonomic dysreflexia. Stimulation of presacral and pelvic nerves is believed to be one of the trigger mechanisms for autonomic dysreflexia [4-5]. One of the most common of these triggers includes bladder distension [8]. With these stimuli, the nervous impulses travel through the spinal cord through the lateral spinothalamic tracts and dorsal columns, which then precipitate an uninhibited sympathetic response through the intermediolateral gray matter [5]. This can manifest as vasoconstriction and severe hypertension, which is then perceived by the carotid sinus and aortic body baroreceptors to generate a vagal response [5, 8].This can lead to vasodilation above the injury, followed by a subsequent reflex of vasomotor parasympathetic bradycardia [8]. However, bradycardia is not always seen and, at times, patients may even present with tachycardia during some episodes [8]. Lumbar spine instability is a rare trigger of autonomic dysreflexia; however, in our patient’s case, the spinal instability at L5-S1 was significant enough to cause the patient’s symptoms.

Autonomic dysreflexia has been reported to be more prevalent in the first few years post injury with decreasing prevalence after 15-20 years in paraplegics [4]. Our case is unusual in that the autonomic dysreflexia occurred over 40 years after the initial injury. However, one must keep in mind that patients with Charcot spine often require more than 10 years to progressively develop spinal instability severe enough to cause symptoms [4]. When managing patients who present with a lesion above T6 and complain of symptoms suggestive of autonomic dysreflexia, one should check imaging of the thoracolumbar spine in addition to the site of injury [4].

Diagnosis of Charcot arthropathy is made by using the clinical picture along with appropriate imaging. Charcot spine’s progressive involvement of vertebral bodies with a soft tissue inflammatory reaction can make the lesion appear similar to a tumor or infection [2, 7]. In such cases, percutaneous vertebral biopsy should be performed to obtain diagnosis prior to performing surgical stabilization [2, 7]. The typical pathology seen in neuropathic arthropathy is bony fragmentation, fibrosis, fibrocartilate, and granulation tissue without evidence of infection or malignancy [2].

Immobilization of the Charcot joint is the most effective way to treat Charcot arthropathy and deter neurologic sequela. Orthotic bracing and activity restriction is sometimes offered to patients who cannot tolerate surgery for various reasons; however, surgical stabilization remains the treatment of choice [3-4,7]. Previous reports of Charcot arthropathy in spinal cord injury patients that has been associated with autonomic dysreflexia have detailed their surgical interventions aimed at repairing the vertebral column [2, 7, 9-10].

The aim of surgery involves debriding inflammatory tissue in order to prepare the site for bony fusion, decompressing spinal stenosis, and obtaining a solid circumferential fusion to stabilize the involved spinal segment [1, 3]. An anterior approach is usually favored for debridement purposes [3]. However, it is insufficient by itself and posterior approach is usually necessary for mechanical stabilization. Many authors favor a combined approach [1, 7].

However, as in our case, a single posterior phase with interbody fusion may be employed in certain cases. Patients with complete sensorimotor paraplegia in particular may benefit from posterior stabilization without the added morbidity that can be associated with an anterior approach [1, 4, 6]. In a series by Delvin, et al. of patients with neuropathic spinal arthropathy, combined anterior and posterior approach was recommended for nonreducible rigid deformity or multiple-level Charcot involvement, while bone grafting of the anterior defect via a posterolateral approach only was favored for other cases [2]. The extent of bony destruction should also be considered when planning surgical approach, as extensive anterior and posterior bony destruction decreases bone stock available for fusion [2]. Thomason, et al. also reported a case in which they achieved a single-stage posterior approach with shortening osteotomy to achieve end-to-end apposition of adjacent vertebral bodies and to reduce kyphosis [6]. All previously fused segments should be incorporated in order to avoid adjacent level pseudoarthrosis and instability at adjacent unfused segments [2-3, 6]. When spanning the lumbopelvic junction, a four-rod construct may be advantageous in select cases over the more commonly used two-rod construct in that it can provide improved stability in flexion-extension and, when cross-linked, in axial rotation [9-10]. The surgical technique in our case similarly utilized a four-rod construct from a posterior approach spanning from T8 to L5 in order to achieve maximum stability while minimizing risk of hardware failure. 

Autonomic dysreflexia associated with Charcot spine is very uncommon. In a literature review by Morita, et al., only five cases were previously reported in addition to the one case that they reported. Autonomic dysreflexia associated with Charcot arthropathy of the lumbar spine is even more rare. There have been a limited number of reports that have shown that surgical stabilization can lead to complete amelioration of autonomic dysreflexia associated with Charcot arthropathy. Our case, using an alternative four-rod construct, strengthens the recommendation that surgical stabilization of an affected lesion should be recommended for these patients. 

## Conclusions

We present one such unusual case of Charcot arthropathy of the lumbar spine causing autonomic dysreflexia and also demonstrate the effectiveness of our approach with limited invasiveness compared to many other reported cases. The results of the surgery were effective immediately in relieving the patient’s symptoms of dysautonomia, and our patient continued to have relief after six months of follow-up.
